# Phenotypes for general behavior, activity, and body temperature in 3q29 deletion model mice

**DOI:** 10.1038/s41398-023-02679-w

**Published:** 2024-03-07

**Authors:** Daisuke Mori, Ryosuke Ikeda, Masahito Sawahata, Sho Yamaguchi, Akiko Kodama, Takashi Hirao, Yuko Arioka, Hiroki Okumura, Chihiro Inami, Toshiaki Suzuki, Yu Hayashi, Hidekazu Kato, Yoshihiro Nawa, Seiko Miyata, Hiroki Kimura, Itaru Kushima, Branko Aleksic, Hiroyuki Mizoguchi, Taku Nagai, Takanobu Nakazawa, Ryota Hashimoto, Kozo Kaibuchi, Kazuhiko Kume, Kiyofumi Yamada, Norio Ozaki

**Affiliations:** 1 Department of Psychiatry, Nagoya University Graduate School of Medicine, Nagoya, Japan; 2 Brain and Mind Research Center, Nagoya University, Nagoya, Japan; 3 Department of Pathophysiology of Mental Disorders, Nagoya University Graduate School of Medicine, Nagoya, Aichi Japan; 4 Department of Neuropsychopharmacology and Hospital Pharmacy, Nagoya University, Graduate School of Medicine, Nagoya, Japan; 5 Department of Applied Pharmacology, Graduate School of Medicine and Pharmaceutical Sciences, University of Toyama, Toyama, Japan; 6 Department of Neuropharmacology, Graduate School of Pharmaceutical Sciences, Nagoya City University, Nagoya, Japan; 7 Center for Advanced Medicine and Clinical Research, Nagoya University Hospital, Nagoya, Japan; 8 Medical Genomics Center, Nagoya University Hospital, Nagoya, Japan; 9 Division of Behavioral Neuropharmacology, International Center for Brain Science (ICBS), Fujita Health University, Toyoake, Japan; 10 Laboratory of Molecular Biology, Department of Bioscience, Graduate School of Life Sciences, Tokyo University of Agriculture, Tokyo, Japan; 11 Department of Pathology of Mental Diseases, National Institute of Mental Health, National Center of Neurology and Psychiatry, Tokyo, Japan; 12 Division of Cell Biology, International Center for Brain Science, Fujita Health University, 1-98 Dengakugakubo, Kusukake-cho, Toyoake, Aichi Japan; 13 Institute for Glyco-core Research (iGCORE), Nagoya University, Furo-cho, Chikusa-ku, Nagoya, Japan

**Keywords:** Molecular neuroscience, Pathogenesis

## Abstract

Whole genome analysis has identified rare copy number variations (CNV) that are strongly involved in the pathogenesis of psychiatric disorders, and 3q29 deletion has been found to have the largest effect size. The 3q29 deletion mice model (3q29-del mice) has been established as a good pathological model for schizophrenia based on phenotypic analysis; however, circadian rhythm and sleep, which are also closely related to neuropsychiatric disorders, have not been investigated. In this study, our aims were to reevaluate the pathogenesis of 3q29-del by recreating model mice and analyzing their behavior and to identify novel new insights into the temporal activity and temperature fluctuations of the mouse model using a recently developed small implantable accelerometer chip, Nano-tag. We generated 3q29-del mice using genome editing technology and reevaluated common behavioral phenotypes. We next implanted Nano-tag in the abdominal cavity of mice for continuous measurements of long-time activity and body temperature. Our model mice exhibited weight loss similar to that of other mice reported previously. A general behavioral battery test in the model mice revealed phenotypes similar to those observed in mouse models of schizophrenia, including increased rearing frequency. Intraperitoneal implantation of Nano-tag, a miniature acceleration sensor, resulted in hypersensitive and rapid increases in the activity and body temperature of 3q29-del mice upon switching to lights-off condition. Similar to the 3q29-del mice reported previously, these mice are a promising model animals for schizophrenia. Successive quantitative analysis may provide results that could help in treating sleep disorders closely associated with neuropsychiatric disorders.

## Introduction

Schizophrenia is a psychiatric disorder that is significantly influenced by genetic factors [1]. However, the pathophysiology leading to the fundamental diagnosis and treatment of this disease remains yet to be elucidated [2, 3]. A major reason for this issue is that although several animal models mimicking human schizophrenia have been developed, no reliable animal model that can be directly used for drug discovery has been established [4–6]. Numerous animal models have been developed that mimic genetically validated rare mutants that are believed to be strongly involved in the pathogenesis of psychiatric disorders [7]. Studies show that phenotypic analysis of model mice may help in elucidating the pathophysiology of psychiatric disorders and developing fundamental psychiatric treatment methods [5, 8, 9].

The 3q29 deletion, along with 22q11.2 deletion, is known to be the highest genetic risk factor for schizophrenia, and several patients have been identified in our copy number variation (CNV) analysis [10–12]. Individuals with 3q29-del show extremely diverse symptoms, including heart disease, immune disorders, and developmental disorders, as well as symptoms of psychiatric disorders [13]. The human 3q29 region contains 25 genes, for example, *Dlg1* and *Pak2*, and when the amount of these gene products is reduced, various organs become susceptible to loss of function. Patients with 3q29-del have risk factors in all organs of the body, including the brain.

The development of genome editing technology has enabled the creation of mouse models that mimic human CNVs. There are two representative reports published on 3q29-del mice. Rutkowski et al. generated 3q29-del mice and observed behavioral abnormalities such as decreased sociality, spatial learning ability, and memory in male mice [14]. Takemoto et al. and Baba et al. independently generated 3q29-del mice and detected behavioral abnormalities such as increased anxiety-like behavior, decreased sociality, and decreased prepulse inhibition (PPI) [15, 16]. Patients with 22q11.2 deletion present with several individual symptoms, and the phenotypes of 22q11.2 deletion model mice vary not only between different groups of mice but also within a single strain [17, 18]. 3q29 deletion syndrome is another disorder that is characterized by a large individual variation. We have created a new 3q29 deletion mouse model that mimics the 22q11.2 deletion and tested numerous schizophrenia-like behaviors to determine robust phenotypes in this model.

Our 3q29-deficient mouse model also presented with underweight and delayed physical growth, which is consistent with the findings of previous studies [14–16]. Moreover, we reevaluated our animal model of schizophrenia by performing a series of general behavioral tests and analyzing the results with those of previous studies. Furthermore, the phenomenon of PV-positive neurons as observed in the mouse model created by Baba et al. was reproduced in the present study [16].

Furthermore, we explored nocturnal activity in this mouse model. Mice are nocturnal animals by nature; however, behavioral analysis is conducted only during the day for human convenience [19], Therefore, to evaluate the activity of mice during the nocturnal activity period, we established a system to measure the activity and body temperature of multiple mice by implanting a small activity sensor, Nano-tag, into their abdominal cavity and acquired data under free-ranging conditions for up to 8 weeks. To date, only a few studies have analyzed this small sensor chip [20, 21]. This chip provides a simple experimental system for understanding whether objective data are related to circadian rhythm abnormalities and sleep disorders, which are believed to be closely associated with psychiatric disorders [22]. Indicators such as activity levels and body temperature can be easily compared between humans and animals and may be worth establishing as a new evaluation method for psychiatric disease models.

## Results

### Generation of 3q29-del mouse model

**Fig. 1:**
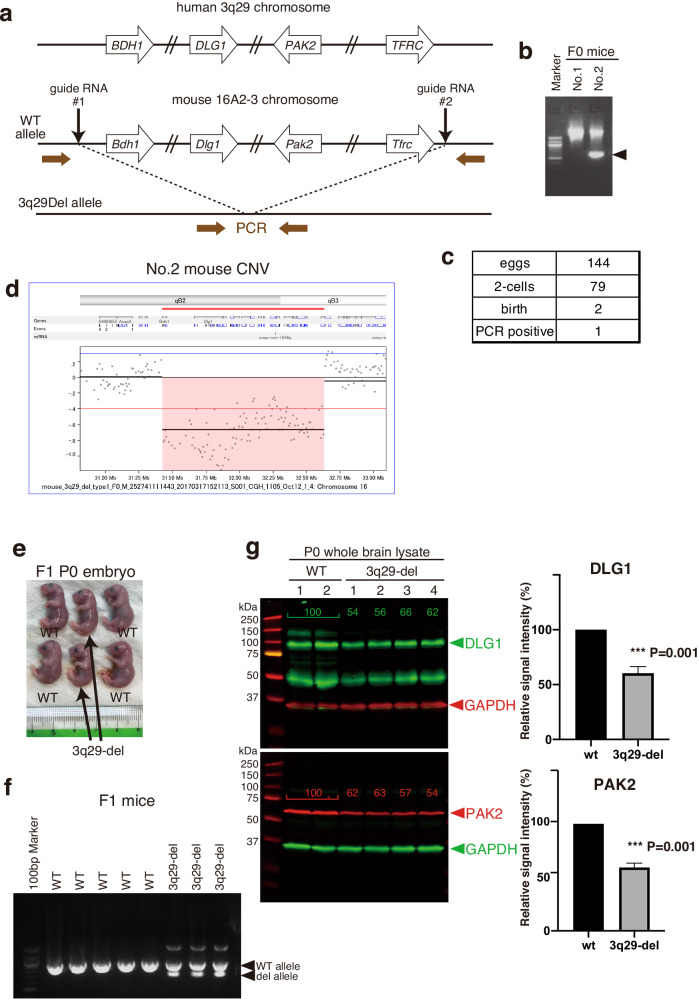
Generation of *3q29*-del mice by CRISPR/Cas9. **a** Structure of the human 3q29 microchromosomal region and its homologous mouse 16B2-3 region and strategies for generating mouse models of 3q29 deletion. **b**, **c** Mixture of two guide RNAs, Cas9 protein, and donor DNA were injected into the pronucleus of mouse fertilized eggs, and genotyping PCR was performed on the two F0 embryos. **d** Genomic DNA was prepared from the tail of No. 2 mouse in (**b**), and deletion of a region corresponding to the human 3q29 microchromosomal region was confirmed using array CGH for mouse. No. 2 mouse in (**b**) was a male, and the litter obtained by mating with female mice of the C57BL/6J strain (**e**); deletion of the *Tfrc* region from *Bdh1* was also confirmed in the F1 pups by PCR (**f**). **g** Whole brain lysates were prepared from whole brains of 3q29-del and WT mice immediately after birth and immunoblotted with antibodies against DLG and PAK2, genes within the 3q29 region. The relative protein levels of both DLG1 and PAK2 were reduced by about half in 3q29-del mouse brains. P0 mouse brains were obtained from WT and 3q29-del, respectively, and DLG1 or PAK2 signals were calculated relative to GAPDH as an internal standard, and both were significantly downregulated in 3q29-del (two-tailed t-test).

To clarify the brain pathology caused by the deletion of the human chromosome 3q29 region, we generated a mouse model of human 3q29-del by genome editing technology. The human chromosome 3q29 region, which contains 25 genes, including *BDH1* and *TFRC*, is equivalent to the mouse chromosome 16B2 region, which also consists of 25 genes, including *Bdh1* and *Tfrc*; we designed mice that lacks this region (Fig. 1a). The CRISPR/Cas9 method was applied to 144 fertilized eggs, and two pups were obtained, one of which was confirmed by polymerase chain reaction (PCR) to possess the deletion allele as designed (Fig. 1b, c). We extracted genomic DNA from the mice and performed whole genome analysis, which confirmed that the CNV deletion existed only in the 16B2 region (Fig. 1d). The offspring from this male mouse were normal (Fig. 1e), and we could detect the deletion mutant allele by PCR (Fig. 1f). Quantification of DLG1 and PAK2 proteins in the 3q29 region using the lysates of fetal brains immediately after birth showed that the expression levels of both genes were half of that of wild-type (WT) brains in the heterozygous deletion (3q29-del) mouse brain (Fig. 1g).

### 3q29-del have low body weight

**Fig. 2:**
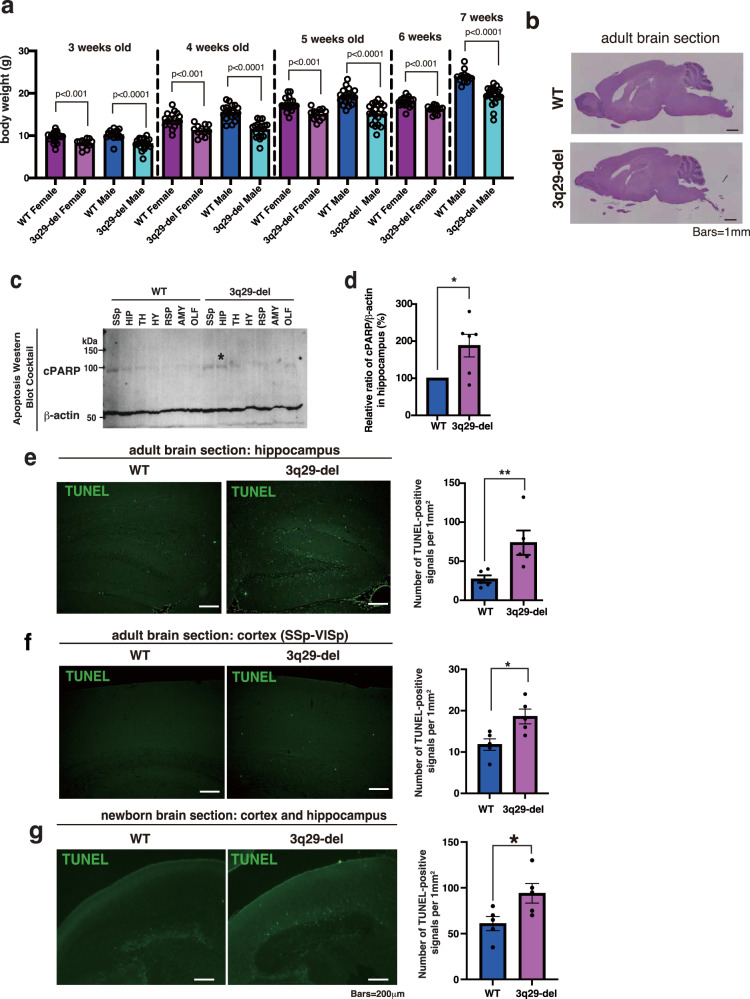
Physical characteristics and histopathological analysis of 3q29-del mice. **a** Sperm from mature 3q29-del males were artificially inseminated with oocytes from the C57BL/6J strain and implanted into sham-pregnant ICR strains. The pups were weighed every other week from 3 to 7 weeks of age, respectively. The Mann–Whitney U test showed significant differences in the weights of pups of both sexes and at all ages. **b** HE staining in the sagittal section of the brain of 16-week-old 3q29-del. **c** The immunoblot analysis for the quantification of 3q29-del brain apoptosis. Several areas of the adult brains were isolated under a stereomicroscope and prepared as total tissue lysate. Subsequently, 20 μg of protein was applied into each well. Cleaved PARP were detected using the apoptosis western blot cocktail. β-Actin was co-detected as an internal control. **d** For each brain region, the ratio of the signal intensity of cleaved PARP corrected for 3q29-del β-actin to WT was calculated and tested using the Mann–Whitney *t*-test. **e**, **f** TUNEL staining of the sagittal sections of the adult 3q29-del and WT mice. TUNEL-positive signals in the hippocampus (**e**) and cortex (mainly primary somatosensory cortex) (**f**) were measured and compared in terms of the number of signals per millimeter square. **g** The sagittal sections of the brains of the 3q29-del and WT mice immediately after birth were stained with TUNEL. Moreover, the TUNEL signals in the hippocampal and cortical regions were measured, and the number of signals per millimeter square was compared between the 3q29-del and WT mice. The bar indicates 200 μm. All TUNEL assays were tested for significance using the Mann–Whitney t-test.

We measured body weight changes during the development of 3q29-del mice and performed general behavioral phenotyping after 8 weeks of age. At 3, 4, and 5 weeks of age, the 3q29-del model mice exhibited lower body weight than the WT mice in the same litter (Fig. 2a). Both sexually mature 6-week-old female and 7-week-old male 3q29-del mice also showed lower body weight than WT mice (Fig. 2a). Hematoxylin and eosin staining revealed no significant structural abnormalities in the adult 3q29-del brain (Fig. 2b). The brain weight of 3q29-del mice was also reduced when compared with that of WT mice (Supplementary Fig. 1a–d).

As apoptosis is important for brain development and formation and has been implicated in SCZ [23, 24], immunoblotting was performed using brain samples of 3q29-del mice. Poly(ADP-ribose)polymerase-1 (PARP-1) is a nuclear molecule implicated in various stress responses, and its cleavage by caspase-3 (cPARP) has been shown to enhance apoptosis [25]. We quantified the amount of cPARP in each brain region of the adult WT and 3q29-del mice, indicating that the amount of cPARP was significantly increased only in the hippocampus among the 3q29-del mice compared with the WT mice (*p* = 0.0476, Mann–Whitney U test) (Fig. 2c, d). Subsequently, we performed TUNEL assays in the hippocampus and cortex of the 3q29-del and WT mice. In the adult hippocampus, apoptosis was increased with an average of 23 signals (*N* = 5) and 59 signals (*N* = 5) in 3q29-del per square millimeter, suggesting that apoptosis was also significantly enhanced in the hippocampus of 3q29-del mouse (*p* = 0.0079) (Fig. 2e). In the adult cortex, TUNEL-positive signals were increased by an average of 12 signals in the WT mice and 18 signals in the 3q29-del mice (*p* = 0.024) (Fig. 2f). Regarding the visual fields of the hippocampus and cortex at birth, TUNEL-positive signals were significantly increased in the 3q29-del mice than in the WT mice (median of WT mice, 13 (*N* = 5); median of 3q29-del mice, 19 (*N* = 5); *p* = 0. 040) (Fig. 2g). To further investigate the histological pathology, extensive immunostaining was performed on the brain of 3q29 deletion mice using markers for astrocytes, oligodendrocytes, and microglia, but no significant differences were observed in any marker or region (Supplementary Fig. 2a–c).

### Histopathological analysis of 3q29-del

**Fig. 3:**
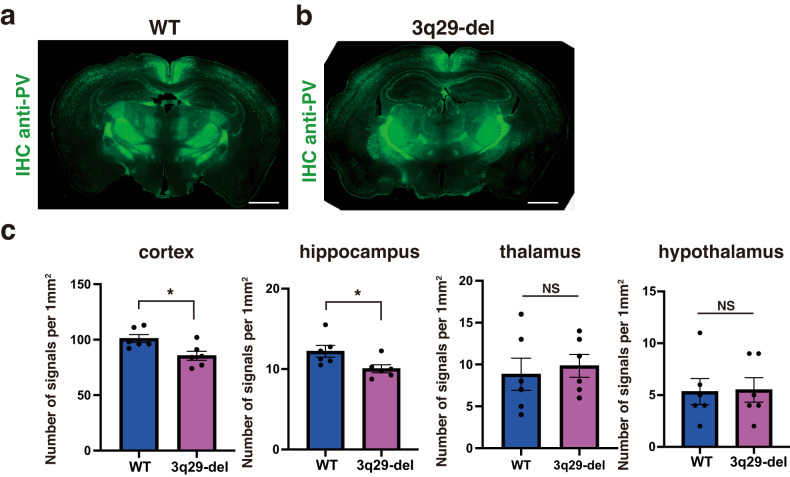
Pathological analysis of PV neurons constituting the 3q29-del brain via immunostaining. The immunohistochemistry of the brains of adult 3q29-del and WT mice. The whole brains of the mice were thinly sliced (coronal) and immunostained with anti-Parvalbumin (PV) antibodies. The fluorescence of the whole sections was observed, and the granular signals were measured in four regions with the help of Fiji (Image J2): cortex, hippocampus, thalamus, and hypothalamus. Furthermore, the number of signals per mm2 was compared between the WT (**a**) and 3q29-del (**b**) mice, and statistical analysis was performed using Student’s *t*-test (**c**). Bars = 1 mm.

The histological comparison of the brains of 3q29-del and WT mice was performed via immunostaining with several neuronal or glial cell markers. In a previous study, the number of parvalbumin (PV)-positive neurons in the cortex of 3q29-del mice was found to be low [16]. Subsequently, we reevaluated the number of PV neurons in the cortex and hippocampus of the reconstituted 3q29-del mice. Moreover, the 3q29-del mice had significantly fewer signals than the WT mice, both in the cortex and hippocampus (hippocampus, *p* = 0.040; cortex, *p* = 0.032; Mann–Whitney U test) (Fig. 3). For further histopathological analysis, the distribution of glial cells was also extensively analyzed. The number of signals per square millimeter between the 3q29-del and WT mice were compared via immunostaining with marker antibodies for astrocytes, oligodendrocytes, and microglia compared, but no significant differences were observed in any of the antibodies or regions (Supplementary Fig. 2). Furthermore, the number of signals per square millimeter in the WT and 3q29-del mice were compared via immunostaining with several neuron-specific antibodies, i.e., NeuN, Arc, and TH, but no significant differences were observed in any of the antibodies or regions (Supplementary Fig. 3).

Mouse models of schizophrenia often exhibit reduced neurite outgrowth in in vitro-cultured neurons [26]. We observed in vitro neurite outgrowth of fetal cerebral cortical neurons prepared from 3q29-del mice by time-lapse imaging, but no significant difference was found (Supplementary Fig. 4).

### General behavioral analysis of 3q29-del model mice

**Fig. 4:**
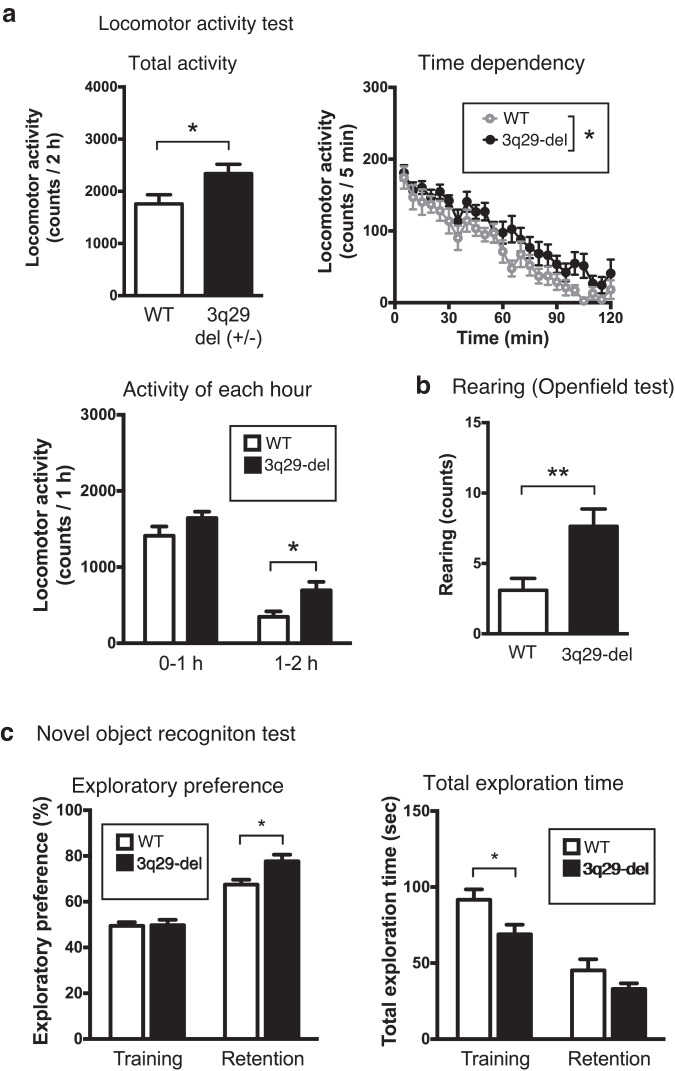
General behavioral analysis of 3q29-del model mice. **a** Result of locomotor activity test. Total activity over a 2 h measurement period (top left), activity every 5 min (top right), activity divided into the first half hour and the second half hour (center left). **b** Comparison of the number of rearing observed under free movement of mice in the open field test. **c** Result of novel object recognition test. Data represent the mean ± SEM (*N* = 11 for WT male mice, *N* = 12 for WT female mice, *N* = 11 for 3q29-del male mice, *N* = 12 for 3q29-del female mice). Refer also Table 1 and Supplementary Fig. 2.

To clarify the pathophysiological significance of 3q29-del mice in vivo, we conducted a series of general behavioral tests on 3q29-del male mice and their WT male littermates. In the spontaneous locomotion test, in which the number of times a mouse crossed an infrared sensor was counted, the activity of 3q29-del mice was significantly higher than that of WT mice after 2 h of measurement, and the difference was more pronounced in the latter hour (Fig. 4a). In the open field test, the frequency of rearing was significantly higher in 3q29-del mice (Fig. 4b). The novel object recognition test showed that 3q29-del mice were more interested in novel objects than WT mice (Fig. 4c, left). The search time was shorter in 3q29-del mice than in WT mice, suggesting an improvement in learning ability (Fig. 4c, right). The results of other behavioral analyses, such as the Y-maze test performed on male mice, were not significantly different (Supplementary Fig. 5a–g). Behavioral analysis of the same parameters in female mice of a different lot (Supplementary Fig. 6) demonstrated that they spent more time in the inner circle of the open field test, suggesting a decrease in anxiety behavior (Supplementary Fig. 6b). Furthermore, we observed in total exploration time in the retention session of novel object for female mice, suggesting a decrease in search time due to improved learning ability (Supplementary Fig. 6g). The results of the behavioral analysis conducted on male and female mice in this study and a comparison with previously reported results are summarized in Table 1.

**Table 1:** Summary of general behavior analysis.

	Mori et al., in this study		Baba et al., NPP, [16]	Rutkowski et al., MP, [14]
	♂	♀	♂	♂♀
Open field test	-	↑(stay in center)	↓(stay in center)	-
Rearing (Open field test)	↑	-	ND	ND
Y-maze test	-	-	ND	ND
Elevated plus maze	-	-	ND	-
Locomotor activity test	↑	-	ND	-
Novel object recognition test	↑	-	ND	ND
Exploration time (NORT)	↓ (Training)	↓ (Retention)	ND	ND
Social interaction test	-	-	↓	↓
Prepulse inhibition test	-	-	↓	-
Strartle response	-	-	↑	↑(female)
Rota-rod test	-	-	ND	ND
Fear conditioning test	-	-	↓	↑(female)
Morris water maze	ND	ND	ND	↓(male)
Marble-burying	ND	ND	ND	-
Amphetamine administrated Locomotor activity test	ND	ND	ND	↓
Risperidone administrated Prepulse inhibition test	ND	ND	↑	ND
Body weight	↓	↓	↓	↓

- : Not changed; ↑: Increase or improved behavior; ↓: Decrease or impaired behavior.

*ND* not done.

### 3q29-del model mice are hypersensitive to lights on and lights off switching

**Fig. 5:**
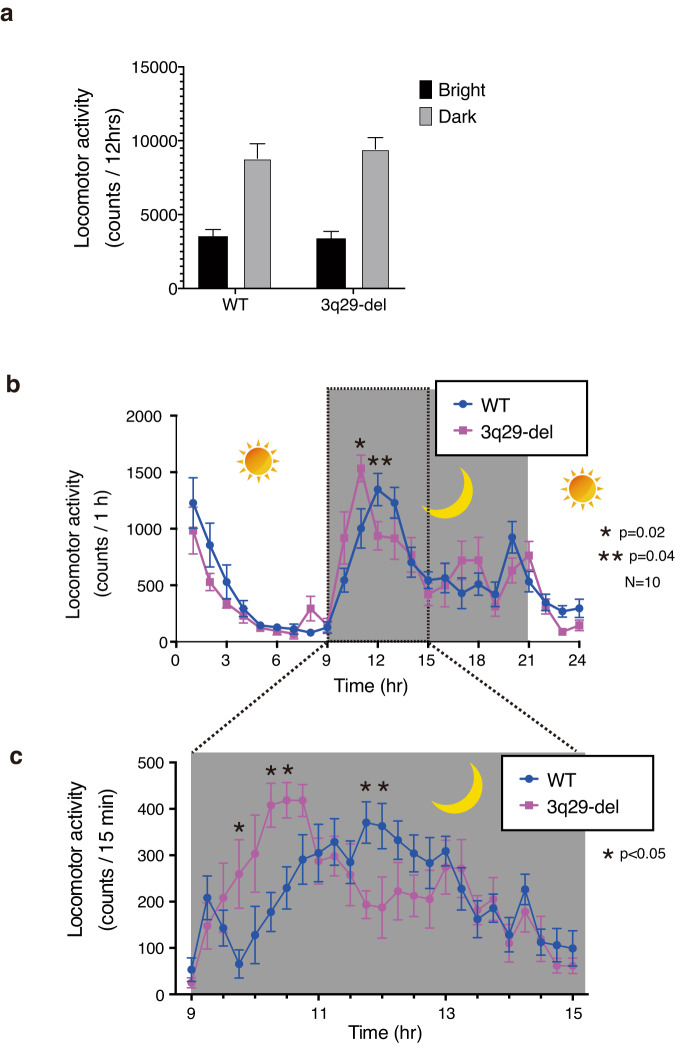
24-h locomotor activity test. **a** Total activity in 24 h (WT mice: *N* = 10; 3q29-del mice: *N* = 10). **b** Graph of activity plotted hourly for the results in (**a**). Indicated as mean ± SE of 10 animals. Multiple *t*-tests were performed at each time period. **c** Graph of activity every 15 min for the 6 h after the switch from light-on to light-off in the measurement in (**b**).

Among a series of general behavioral tests, the results of the locomotor activity test suggested that 3q29-del mice were hyperactive. Because this test was performed for a short period of time (2 h after a brief acclimation), we measured the activity for a longer period of time (24 h) to account for the diurnal nature of the test. As in the previous locomotor activity tests in behavioral testing, we obtained data for 3q29-del mice and WT mice at 10 weeks of age. The 24-h locomotor activity test was conducted after habituation to 9-h lights, 12-h lights off, and 3-h lights on. Unlike the results of tests completed in 2 h of daytime (Fig. 4a), there was no significant difference in the total amount of activity for each group (Fig. 5a), probably due to the total length of the measurement time. We next plotted the hourly activity volume against time. Data for individual mice are shown in Supplementary Fig. 7. The average value from these time points showed that the increase in activity immediately after lights off was more rapid in 3q29-del mice than in WT mice (Fig. 5b); even for the 15-min interval data, the 3q29-del mice exhibited a significantly faster increase in activity after lights off than the WT mice (Fig. 5c).

### Diurnality of long-term activity and body temperature measurements in 3q29-del model mice using Nano-tag

**Fig. 6:**
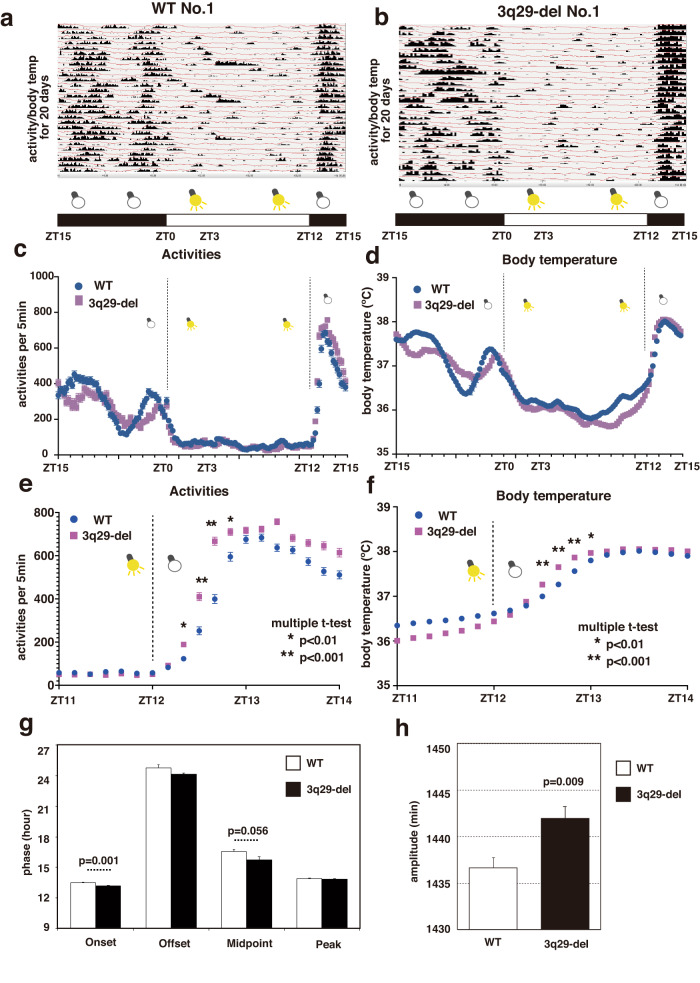
Long-term activity and body temperature measurement with a miniature accelerometer: Nano-tag. **a**, **b** The data are from the L-D cycle of a mouse model in which the Nano-Tag was surgically implanted intraperitoneally and displayed by the Nano-Tag Viewer software. Data for the first 20 days are shown. The horizontal axis is the time axis from ZT15 (AM0) to ZT15 (PM12) of the next day, the histograms on the vertical axis show the amount of activity for each 5 min, and the line graphs show the body temperature; (**a**) WT, (**b**) 3q29-del mice. Overall data are shown in Supplementary Fig. 4. Mean ± standard error of 6-week data from five animals each of (**c**) WT (**d**) 3q29-del measured in the L-D cycle plotted by time. The vertical axis shows activity in 5 min and the horizontal axis shows time in twenty-four hrs. **e**, **f** Enlarged views from the data in (**c**) and (**d**) for the 3 h from ZT11 to ZT14. **g** Activity phase analysis under L-D cycle conditions [53]. The time at which the 24 h moving average activity level exceeded the 3 h moving average activity level was defined as the onset of activity (onset) and the time at which it fell below (offset). The middle point was defined as the point at which the cumulative amount of activity from the start of the activity was half of the total amount of activity for the entire period (16 h). The time of the maximum value of the 1-h moving average was set as (peak). Statistical analysis was performed with a two-tailed *t*-test and was significant for onset (*p* = 0.001). **h** Circadian cycle determined by FFT analysis from each individual’s data in the D-D cycle for 13 days. According to t-test, 3q29-del was 5.3 min longer than WT, and the difference from WT was significant (*p* = 0.009).

In the 1-day locomotion test in which 3q29-del male mice were kept alone, there was an increase in activity after the change from lights on to lights off, which was more sensitive than that in WT mice. We analyzed this observation more quantitatively in a more natural situation. In general, in the locomotor activity test, the amount of activity is measured by the number of times the mouse crosses the infrared ray in the cage, but long-term measurements were not possible in this study because the animal bedding was not laid. To address this problem, we implanted a small acceleration sensor chip for rodents (product name Nano-tag) in the abdominal cavity of 3q29-del female mice and WT female mice. After implantation, the mice were placed in the same cage for each genotype for a healing period of 1 week. Then the mice were acclimated to the light–dark (L–D) cycle, in which the lights were automatically turned off and on every 12 h, after which measurements were started. The histogram revealed a clear trend of increased activity when the lights were off and decreased activity when the lights were on; hence, we concluded that the measurement system was working well (Fig. 6a–b, Supplementary Fig. 8). We obtained activity and body temperature data from all mice for 6 weeks in the L–D cycle. Similar to the 24-h locomotor activity test, the 3q29-del mice exhibited a significantly faster increase in activity when the lights were off (Fig. 6c, e) and a faster increase in body temperature (Fig. 6d, f) than the WT mice. We also attempted to conduct this experiment with male mice, but it was difficult to obtain stable data due to the high frequency of fights caused by group rearing (data not shown).

## Discussion

The 3q29-del mouse model has already been developed and behaviorally analyzed by several research groups, and we performed a comparison of results of these studies, as shown in Table 1 [14–16, 27]. In our analysis, we observed an increase in rearing frequency in the open field test, an increase in locomotor activity, and an increase in the novel object recognition test. However, we found no decreases in social interaction or PPI, which is slightly different from previous studies. All mice were generated from the C57BL/6J strain, and the differences in the DNA sequence of the 3q29-del region were minor and well validated. There are also no obvious differences in behavioral phenotypes. Our 3q29-del mice harbored no CNVs other than 16B2 as assessed by whole genome analysis (Fig. 1d). Therefore, we believe that our design of the 3q29 deletion model was quite reasonable. It is possible that delicate behavioral phenotypes are significantly affected by differences in the raising environment and the assessor, and the results may vary. However, the 3q29-del mice were consistent in their phenotype of weight loss [14, 16].

3q29-deficient mice have already been developed and behaviorally analyzed by several research groups including ours as summarized in Table 1 [14–16]. Although the behavioral phenotypic comparisons between mouse models are difficult to conduct owing to the differences in the statistical procedures in each study, the results are still different from those of previous studies, with no reduction in social interactions or PPI. In the present study, in the 3q29-del mice, Cas9 protein and guide RNA were microinjected into the fertilized egg pronucleus of the C57BL/6J strain, which was cleaved at two sites with cis, while the cleaved sites were connected and repaired, and the connections were confirmed by sequencing. The 3q29-del mice in the present study harbored no CNVs other than 16B2 as assessed via whole genome analysis (Fig. 1d). In the study of Rutkowski et al., their mouse model was generated by the microinjection of Cas9 mRNA and guide RNA into the fertilized egg pronuclei of the C57BL/6N strain, and the cleavage site by guide RNA was different from ours. In the mouse model of Baba et al., loxP sequences were knocked into the target site by introducing a Cas9 plasmid and guide RNA into the embryonic stem (ES) cells of the C57BL/6J strain, and the recombinant ES cells were injected into the blastocysts through the transient expression of Cre recombinase to produce the chimeric mice. Thus, they do not have completely identical deletions due to the differences in strain and method of generation. Considering this, it can be suggested that the sensitive behavioral phenotype may be greatly affected by the differences in the breeding environment and evaluators, in addition to the differences in the sequences among the mouse models. However, the most objective phenotype of 3q29-deficient mice being underweight is very robust, and either mouse model would be equally valid [14, 16].

This study also evaluated the pathological abnormalities in the brain of 3q29-deficient mice. During apoptosis, the major regions of the brain were most enhanced in the hippocampus and cerebral cortex, and a decrease in the PV neurons as a neuronal cell type was observed, which is consistent with the findings of Baba et al. It can be concluded that the pathological phenotypic abnormalities may also be robust in the brain of 3q29-del mice immediately after birth. The possibility that apoptosis may occur during the developmental period, as apoptosis was found to be enhanced in the brain immediately after birth, should be considered more carefully in terms of susceptible time periods, regions, and cell types.

3q29 deletion is one of the largest risk factors for schizophrenia [28]. In addition to psychiatric symptoms such as schizophrenia, various systemic disorders such as low birth weight or failure to thrive, short stature, ataxia, abnormal skull shape, and heart defects have been reported as characteristics of patients with chromosome 3q29 deletion. Therefore, early diagnosis during the developmental process and long-term follow-up are essential [29–31]. In this context, case reports of patients who have been followed up since childhood show that all patients have severe treatment-resistant schizophrenia; and mild-to-moderate learning disabilities, developmental delay, facial dysmorphism, and microcephaly have been observed [29, 30]. In our mouse model mimicking the 3q29 deletion, no phenotype similar to the human case has apparently emerged, which may be affected by the genetic background of the mouse, although this must be investigated in more detail in the future. However, the behavioral phenotype was reconfirmed, strengthening the efficacy of the mouse as a model for schizophrenia. Hence, the possibility of early diagnosis and drug discovery based on the phenotype of these mice has increased.

To achieve this goal, it is important to elucidate the molecular pathogenesis. DLG1 has been identified as a molecule localized in the presynaptic nerve endings of excitatory synapses [32–34], and the postmortem brain analysis of patients with schizophrenia showed that DLG1 protein expression in the prefrontal cortex was reduced to less than half of that in controls while the expression of the interacting molecule GluR1 was also reduced [35]. E17.5-day embryos of DLG1 homo-deleted mice exhibited lower body weights than WT mice and mice with heterozygous deletions [36]. PAK2 is a kinase that is activated by Rac1 and Cdc42 of the Rho family and is involved in cytoskeletal remodeling and cell dynamics [37], suggesting an association with ASD [38]. The social behavior of mice with PAK2 heterozygous deletion is reduced and may be related to the social behavior previously observed in 3q29-del mice (Table 1) [38]. For DLG1 and PAK2, we observed that the protein levels were approximately halved in the brain of 3q29-del mice (Fig. 1g). Other genes in the 16B2 region also have vital roles in the brain. For instance, *FBXO45* is present at both presynapses and postsynapses, regulates ubiquitin-dependent protein degradation systems, and is involved in synapse function [39]. Moreover, rare variants in *Fbxo45* have been identified in patients with schizophrenia, suggesting a link to the disease [40].

In the 3q29-del mice generated by Baba et al., the early gene expressing in neurons, such as *c-fos*, was elevated in the auditory cortex compared with that in WT mice, indicating increased neural activity [16]. Thus, *c-fos* mapping of the whole brain of 3q29-del mice may provide clues to elucidate neural circuit pathology; however, it may differ significantly between light and dark phases [41]. In our study, mice in the light phase were obviously less active and hypothermic than those in the dark phase (Fig. 6). We used Nano-tag, a small sensor chip implanted in the body, as a new method to analyze behavioral phenotypes in mouse models of mental illness. The sensor can acquire data on activity and body temperature over a 24-h period for up to 2 months. Currently, the sensor weighs 2 g, which is sufficient for rats [42, 43]. Nevertheless, we cannot deny the fact that this load is quite large compared with the body size of a mouse, which is different from the natural situation. In our analysis, it was difficult to use 8-week-old mice, which are suitable for general behavioral analysis, but only larger, more mature mice aged 5–6 months were used. With more compact sensors, it will be possible to apply the method from a younger stage of mice, and it will also be easier to check and compare with several existing behavioral analyses. With the increasing number of studies using Nano-tag, simultaneous comparisons of activity and temperature data across models of psychiatric disorders could help determine whether the phenotype is unique to the 3q29-del mouse model or is more frequently observed in other models.

There is some information on the interpretation of successive changes in activity and body temperature obtained by introducing Nano-tag into mice. However, we were surprised to find that the diurnal variation of activity was similar to that of the conventional method, in which activity was measured by the number of times the animal crossed the infrared sensor. Furthermore, adding information about body temperature changes has an advantage, and it will be possible to relate it to sleep data. Activity and body temperature data can be obtained by changing the bedding regularly once a week, replenishing food and water, and leaving the rest of the data alone. In addition, the fact that individual identification is possible and that data can be measured in group housing is also a significant advantage in terms of stress reduction. In this study, we used only female mice, so there was almost no noise due to fighting between mice; however, it would be necessary to prevent fighting in male mice. It is known that the rhythms of mice in the dark–dark cycle have a period of every 23.7 h [44], and in our system, we observed that all mice tended to move up the timing from the quiescent to the active phase of their activity. This suggests that the environment was not stressful enough to alter their rhythms (Supplementary Fig. 6). In contrast, in the L–D cycle environment, we observed a significant difference between the two experimental systems, wherein the 3q29-del mice exhibited an earlier transition from quiescence to activity in response to lights off than the WT mice (Fig. 6). The 3q29-del mice may be hypersensitive to light stimuli due to abnormalities in the function of cones and rods, which constitute the retina, or in melanopsin, a photoreceptor [45]. Severe sleep/circadian disruption occurs more frequently in patients with schizophrenia [46]. Additional research on brain regions related to visual pathways is necessary.

Sleep disturbance is a frequently observed medical condition in mental disorders [22, 47]. The accumulation of continuous activity and body temperature data in mouse models of mental disorders, along with the identification of molecular mechanisms and associated neural circuits, may help in investigating sleep disorders, which are closely related to mental disorders [48]. Furthermore, it may be possible to quantitatively evaluate social interactions between mice based on changes in the coordinates of individual mice as they move, although it will be necessary to make significant improvements in the storage capacity and battery capacity of the chip. To verify how this relates to the pathology of patients with 3q29 deletion syndrome, it will be important in the future to match data with patients’ activity and sleep data.

Our newly generated 3q29-del mice, similar to those reported previously, would be a promising animal model for schizophrenia. Activity and body temperature measurements under natural conditions using Nano-tag are simple and could aid in phenotypic analysis that could be easily validated in various neuropsychiatric disease models. Further studies that are focused on rhythm and sleep in 3q29-deficient mice and a better understanding of the molecular pathogenesis of the disease are needed to develop effective therapeutic approaches for sleep disorders that are closely related to neuropsychiatric disorders. Future approaches include transcriptome analysis using the postmortem brains of patients, mice, and patient-derived iPS cells as reported by Sefik et al. to analyze genes within the 3q29 region and phenotypic analysis of as-yet-unknown miRNAs and long non-coding RNAs (lnc RNAs). A phenotypic analysis of genes in the 3q29 region as well as genes deleted from as-yet-unknown miRNAs and long non-coding RNAs (lnc RNAs) should be conducted in consideration of species differences [49].

## Materials and methods

### Animal experiments

All research and animal care procedures were approved by the Nagoya University Animal Care and Use Committee. Mice were housed in groups of maximum six animals per cage and maintained on a regular 12 h light/dark cycle (9:00–21:00 light period) at a constant 23 °C. Food and water were available *ad libitum*.

### Generating *3q29*-del mice by CRISPR/Cas9 system

For the deletion of the region covering the *Bdh1* to *Tfrc* genes of mouse chromosome 16, which corresponds to the human 3q29 region, guide RNAs were designed outside the Bdh1 and Tfrc genes and generated by the CRISPR/Cas9 method. The selection and optimization of the guide RNA was performed according to our previous reports.

For 3q29 deletion model mice production by mycroinjection including Cas9 proteins, 3q29-crRNA and tracrRNA, and donor single-strand DNA (dsDNA) [50–52]. Cas9 proteins were purchased from NEB (M0386S, USA). The mixture was injected into pronuclei of one-cell-stage zygotes obtained from C57BL/6J strain (Charles River, USA). The details are described in Supplementary materials.

### General behavioral analysis

General behavioral analysis of 3q29-del mice was started with open field test at 8 weeks of age. A series of tests were performed with 11 male WT mice, 11 male 3q29-del mice, 12 WT females, and 12 female 3q29-del mice. The details of the experiments are described in Supplementary materials.

Biological data are expressed as the mean ± SE. Differences between two groups were analyzed by two-tailed Student’s *t*-test. Differences in locomotor activity, PPI test, fear conditioning test and Rotarod test were analyzed by repeated analysis of variance (ANOVA). Multiple group comparisons were made by one-way ANOVA, followed by Tukey test when F ratios were significant (*p* < 0.05). The details are described in Supplementary materials.

### Measuring activity and body temperature with a small sensor chip, Nano-tag

The Nano-tag (Kissei Comtec Co. Ltd., Nagano, Japan) apparatus measuring spontaneous locomotive activities and body temperature was implanted in the abdominal cavity of mice. The mice were allowed to recover and acclimate to the L–D cycle for 10 days after surgery. Five mice were housed together in one cage; their bedding was changed, and food and water were provided once a week while the lights were turned on. Locomotor activity and body temperature were recorded every 5 min after the start of the measurement for 6 consecutive weeks. These data were analyzed using the Nano-tag Viewer software (Kissei Comtec Co., Ltd.).

### Supplementary information

Supplementary materials

## Data Availability

The datasets generated during and/or analyzed during the current study are available from the corresponding author upon reasonable request.
