# Supplementary material for: Phenotypes for general behavior, activity, and body temperature in 3q29 deletion model mice
Source: Transl Psychiatry. 2024 Mar 7;14:138. doi: 10.1038/s41398-023-02679-w (PMC10920862; doi:10.1038/s41398-023-02679-w)
Supplement: Supplementary file 1 — Supplementary materials [file 41398_2023_2679_MOESM1_ESM.docx]

**Supporting Information**

**Table of contents**

**I. Supplementary Methods**

**II. Supplementary Figures**

**Figure S1 Brain weight to body weight ratio of 3q29-del mice**

**Figure S2 3q29-del immunostaining of adult mouse brain with glial cell marker antibodies**

**Figure S3 3q29-del immunostaining of adult mouse brain with neuron specific marker antibodies**

**Figure S4 Neurite outgrowth analysis of E15.5 cortical neurons by time-lapse observation**

**Figure S5 General behavioral analysis of 3q29 deletion model male mice**

**Figure S6 General behavioral analysis of 3q29 deletion model female mice**

**Figure S7 Individual data of each mouse for 24-hrs locomotion activity test**

**Figure S8 Successive data on activity and body temperature in mice implanted with Nano-tag in the abdominal cavity**

**III. Supplementary References**

**I. Supplementary Methods**

**T7 endonuclease I assay**

T7 endonuclease I (T7EI) assay using mouse Neuro2a cells was performed as described previously. Briefly, *3q29-*pX458 or empty pX458 plasmid was transfected into Neuro2a cells in a 12-well plate using Lipofectamine 3000 (Life Technologies). At 48 hours post-transfection, genomic DNA was prepared by proteinase K (P2308, Sigma-Aldrich, USA) digestion and ethanol precipitation. Next, *3q29* loci were amplified with PCR from the purified genomic DNA with specific primers (Supplementary Table 2). The PCR products were slowly annealed and digested with the T7EI enzyme at 37°C for 30 minutes and analysed by electrophoresis in 2% agarose gel.

**Chemical synthesis of crRNA, tracrRNA and donor DNA**

*3q29*-CRISPR-RNA (crRNA) (5’- GUGUCUACUUGUGCACACGUguuuuagagcuaugcuguuuug

-3’ and 5’- GCGUAACUACUACUCUGGUGguuuuagagcuaugcuguuuug

-3’) and transactivating RNA (tracrRNA) (5’- AAACAGCAUAGCAAGUUAAAAUAAGGCUAGUCCGUUAUCAACUUGAAAAAGUGGCACCGAGUCGGUGCU-3’) were chemically synthesized and purified by polyacrylamide gel electrophoresis (Fasmac, Atsugi, Kanagawa, Japan). A donor single-strand DNA(ssDNA) (5'- TAAAAAACGGGATTTATTTTCATTTACGTGTTTGTGTCTACTTGTGCACAGAATTCCAGAGTAGTAGTTACGCAGACACACTGATCTCTTGATAGAGATTCTAATC -3' was also chemically synthesized and purified by polyacrylamide gel electrophoresis (Eurofin Genomics, Tokyo, Japan).

**Injection**

Mixtures for injection were prepared according to a previously defined method. To develop 3q29-del mouse, Cas9 proteins, *3q29*-crRNA and tracrRNA and donor ssDNA were mixed in 0.1 M Tris-EDTA buffer to working concentrations of 100 ng/μl, 0.61 pmol/μl, 0.61 pmol/μl, and 10 ng/μl, respectively. Cas9 proteins were purchased from NEB (catalog # M0386S, USA). Donor ssDNA were chemically synthesized by Fasmac (Japan). The mixture was incubated at 37°C for at least 15 minutes and injected into pronuclei of one-cell-stage zygotes obtained from C57BL/6J mice (Charles River, USA).

**PCR screening and genotyping**

For PCR screening of 3q29*-*del embryos born from the zygotes injected with CRISPR mix, genomic DNA was prepared from tails by proteinase K digestion and subsequent phenol extraction using standard methods. 3q29-del mice were screened by PCR with KOD FX Neo (TOYOBO, Japan) and analysed by electrophoresis in 2% agarose gel. PCR products were further cloned with the Zero Blunt TOPO PCR cloning kit (Life Technologies, USA) and analysed by sequencing as described previously.[1](#_ENREF_1) Conventional screening after establishment of the mouse line was performed by PCR using the mixture of three primers below.

3q29_common_FW primer: 5'-GAAGTTAACCATATATTGGAGTCTGGGGG-3',

3q29_Del_RV primer :5'-GAGAATGCTTTGTCTGGAGACAATGATAGG-3',

3q29_WT_RV primer: 5'- GGGTCTCTCCTTCCTGTTCCATCAGGCATG-3'

**Immunoblot analysis**

For immunoblotting, whole-brain extracts were lysed with sodium dodecyl sulphate (SDS) lysis buffer (4% SDS, 20% glycerol and 50 mM Tris-HCl pH 6.8) and sonicated on ice. The lysates were diluted to 5 μg/μl. For loading, equal volumes of 0.01% bromophenol blue and 1M 2-mercaptoethanol were added to the extracts, which were not heat-denatured. A total of 50 μg non-denatured protein was loaded to each well of 5%–20% SDS gradient polyacrylamide gels (e-PAGEL, ATTO, Japan) and transferred to nitrocellulose membranes (Protran, Whatman). The membranes were probed with antibodies against DLG1 (rabbit polyclonal, ab3437, Abcam), PAK2 (rabbit polyclonal, #2608, Cell Signalling) and GAPDH (mouse monoclonal, clone#3H12, MBL, Japan), followed by incubation with appropriate donkey secondary antibodies (IRDye 680RD and 800CW; LI-COR Biosciences). An infra-red imager was used for the detection and quantification of signal intensities (Odyssey, LI-COR Biosciences). Apoptosis Western Blot Cocktail (Abcam, US) and iBright FL1500 (Thermofisher, US) were used to detect apoptotic signals. Digital data were exported in TIFF format.

**Histological analysis with HE staining**

Paraffin-embedded brain tissue sections (5 μm) were deparaffinized and placed in hematoxylin in distilled water for 10 minutes. The sections were rinsed in running tap water, dipped in hot water at 50ºC to remove overstaining, stained with eosin for 2 minutes, dehydrated and mounted.

**TUNEL assay**

Click-iTTM Plus TUNEL Assay Kits for In Situ Apoptosis Detection (Invitorgen, US) was used to detect apoptotic cells in the brain. The kit was used to stain 5-μm-thick paraffin brain sections, and image data were acquired with a BZ-X800 (Keyence, Japan), 4x objective lens, and the signal-positive cells on the tissue were counted. Statistical analysis was performed by Mann-Whitney t-test using Graphpad Prism8 Software.

**Histological analysis by immunostaining**

Adult WT and 3q29-del mice were perfusion-fixed in 4% paraformaldehyde/phosphate buffer, brains were permeabilized with fixative solution overnight, and 50μm coronal sections were prepared with a vibrating blade microtome (Leica Biosystems VT1200, US) Bregma -1mm~-2 mm brain sections were immunostained with the following antibodies: anti-Parvalbmin (PV) (Synaptic Systems (SySy), guinea pig polyclonal antibody), anti-GFAP (SySy, guinea pig monoclonal antibody), anti-Iba1(SySy, guinea pig monoclonal antibody), anti-CNP1(SySy, guinea pig polyclonal antibody), anti-NeuN (Merck, mouse monoclonal antibody), anti-Arc (SySy, rabbit polyclonal antibody), and anti-TH (SySy, guinea pig polyclonal antibody). Secondary antibodies were Alexa488-labeled anti-guinea pig IgG, anti-rabbit IgG or anti-mouse IgG (Thermofisher, US). Fluorescence observation was performed using BZ-X800. Fluorescence observation was performed using BZ-X800 (Keyence, Japan). Whole coronal sections of adult mice were imaged with a 4x objective lens, and the images were concatenated by the built-in software and exported in TIFF file format. Fiji (ImageJ2) was used to analyze the images [2](#_ENREF_2). The cortex, hippocampus, thalamus, and hypothalamus were manually outlined [3](#_ENREF_3). Cells were individually thresholded based on the green channel background, and cells were counted using the Puncta analyzer plugin. The number of signals per unit area was measured by the area of the selected region.

**Animal Experiments**

All research and animal care procedures were approved by the Nagoya University Animal Care and Use Committee. Mice were housed in groups of maximum six animals per cage and maintained on a regular 12 hours light/dark cycle (9:00 to 21:00 light period) at a constant 23°C. Food and water were available ad libitum.

**General Behavioral analysis**

Behavioral tests were performed in the following order: open field test, Y-maze test, elevated plus maze test, locomotor activity test, novel object recognition test, social interaction test, rotarod test, prepulse inhibition test, and fear conditioning test. The heterozygous 3q29-del model mice were maintained by crossing them with C57BL/6J provided by Jackson Laboratory Japan, and more than eight backcrosses were performed. The 3q29-del and WT animals used for behavioral analysis were 8-15 weeks of age.

**Open field test**

In the open field test, mice were placed at the center of an open field (diameter, 60 cm; height, 35 cm) under moderate light conditions (60 lx) and allowed to explore it for 5 minutes, while their activity was measured automatically using the ethovision automated tracking program (Brainscience Idea Co. Ltd., Osaka, Japan). The open field was divided into an inner circle (diameter, 40 cm) and an outer area surrounding the inner circle. The movement of mice was measured via a camera mounted above the open field. Measurements included distance in the inner and outer sections.

**Y-maze test**

The Y-maze test was performed as described previously.[4](#_ENREF_4)^,^ [5](#_ENREF_5) Each arm was 40 cm long, 12 cm high, 3 cm wide at the bottom, and 10 cm wide at the top. The arms converged in an equilateral triangular central area that was 4 cm at its longest axis. Each mouse was placed individually at the center of the apparatus and allowed to move freely through the maze during an 8-minute session. The series of arm entries was recorded visually. Alternation was defined as successive entries into the three arms on overlapping triplet sets. Percent alternation was calculated as the ratio of actual to possible alternations (defined as the total number of arm entries minus 2) multiplied by 100. Spontaneous alternation (%) defined as successive entries into the three arms on overlapping triplet sets was associated with the capacity of working memory.

**Elevated plus maze test**

The elevated plus maze was constructed and conducted as previously described [4](#_ENREF_4)^,^ [5](#_ENREF_5) with minor modifications. The apparatus was made of plastic material and was elevated to a height of 50 cm above the ground. Each arm of the plus maze was 16 cm in length and 10 cm in width. Additionally, the closed arms had wall enclosures that were 20 cm high. The central platform was a square of 10 × 10 cm. Light intensity around the maze was set at 100–120 lx. Mice were placed on the elevated plus maze for 5 minutes. Mice were placed on the elevated plus maze facing the open arm opposite to the experimenter. The number of entries and time spent in the open and closed arms were recorded over the entire duration of the test.

**Locomotor activity test**

In the measurement of locomotor activity, mice were placed individually in a transparent acrylic cage with a black frosted Plexiglas floor (25 × 25 × 20 cm), and locomotor activity was measured every 5 minutes for 120 minutes using digital counters with an infrared sensor (BrainScience Idea, Osaka, Japan). 3q29-del and WT mice were habituated to the test environment for 60 minutes before the measurement of locomotor activity (habituation period).

For 24-hours test, locomotor activity was measured every 5 minutes for 24 hours using digital counters with an infrared sensor (BrainScience Idea, Osaka, Japan). For the first 9 hours (0-9 hours), the lights are on. For the next 12 hours (16-21), the lights are turned off. The last 3 hours (22-24) the lights are turned on again. 3q29-del and WT mice were habituated to the test environment for 120 minutes before the measurement of locomotor activity (habituation period).

**Novel object recognition test**

The novel object recognition test was performed as described previously. [4](#_ENREF_4)^,^ [5](#_ENREF_5) with minor modifications. Mice were individually habituated to an open-box (30 × 30 × 35 cm) for 3 days. During the training session, two novel objects were placed in the open ﬁeld and animals were allowed to explore for 10 minutes under moderate light conditions (15 lx). The time spent exploring each object was recorded. The preference index was calculated as the ratio of time spent exploring one of the objects to the total exploration time. During test sessions, animals were placed back into the same box 24 hours after the training session, one of the familiar objects used during training was replaced by a novel object, and mice were allowed to explore freely for 5 minutes. The preference index in the test session, the ratio of the amount of time spent exploring the novel object over the total time spent exploring both objects, was used to measure cognitive function.

**Social interaction test**

In the social interaction test, we used the experimental paradigm described previously [4](#_ENREF_4)^,^ [5](#_ENREF_5) by to measure sociability and social novelty preference behavior. The behavioral testing apparatus was a black Plexiglas rectangular box (52 cm long × 25 cm wide × 23 cm tall) consisting of three interconnected chambers. The two end chambers were of an equal size (19 cm × 25 cm), and the middle chamber was smaller (12 cm × 25 cm). Two identical clear Plexiglas cylinders (each 7 cm in diameter, 12 cm tall, multiple small holes) were placed in the testing apparatus, one of each ends of the chamber. All sessions were conducted under conditions of illumination (15 lx). In the habituation phase, a test mouse was placed in the chamber and allowed to explore for 10 minutes. In sociability session, an unfamiliar BALB/c mouse (stranger 1) that had no prior contact with the subject mouse was placed in one of the side chambers. The test mouse was allowed to explore the entire social test box for a 10-minute session. In social novelty session, a second, unfamiliar mouse (stranger 2) was placed in the chamber that had been empty during the sociability test. Measurements were taken of the amount of time spent in each zone by the ethovision automated tracking program (Noldus. Wageningen, Netherlands). A zone was defined as the area surrounding the Plexiglas cylinder (diameter of 19 cm).

**Prepulse inhibition test**

The prepulse inhibition (PPI) test was performed as described previously. [4](#_ENREF_4)^,^ [5](#_ENREF_5) After animals were placed in the chamber under moderately bright light conditions (180 lx) (San Diego Instruments, San Diego, CA), they were allowed to habituate for 10 minutes, during which 65-dB background white noise was present. Animals then received 10 startle trials, 10 no-stimulus trials, and 40 PPI trials. The inter-trial interval was between 10 and 20 seconds and the total session lasted 17 minutes. The startle trial consisted of a single 120-dB white noise burst lasting 40 ms. PPI trials consisted of a prepulse (20-ms burst of white noise at an intensity of 69, 73, 77 or 81 dB) followed, 100 ms later, by the startle stimulus (120 dB, 40 ms white noise). Each of the four prepulse trials (69, 73, 77, or 81 dB) was presented 10 times. Sixty different trials were presented pseudo-randomly, ensuring that each trial was presented 10 times and that no two consecutive trials were identical. The resulting movement of the animal in the startle chamber was measured for 100 ms after startle stimulus onset (sampling frequency 1 kHz), rectiﬁed, ampliﬁed, and fed into a computer, which calculated the maximal response over the 100-ms period. Basal startle amplitude was defined as the mean amplitude of 10 startle trials. PPI was calculated according to the formula: 100 × [1 - (PPx/P120)]%, in which PPx was the mean of 10 PPI trials (PP69, PP73, PP75, or PP80) and P120 was the basal startle amplitude.

**Rotarod test**

The rotarod test was performed according to a previous study [4](#_ENREF_4)^,^ [5](#_ENREF_5) with minor modifications. In brief, the rotarod test was performed under moderate light conditions (15 lx). Mice were trained for 3 consecutive days. During this training phase, mice were placed on a rod rotating at 6 revolutions per minute (rpm) and the time taken for them to fall from the rod was measured. If a mouse stayed on the rod until the end of the 2-minute trial, a time of 120 seconds was recorded. The test phase was performed on day 4. Mice were placed on a rod rotating at 12 rpm and the time taken for them to fall from the rod was measured. Each mouse was subjected to 6 trials per day with a 15-minute inter-trial interval in the training and test phases. We calculated the average value in a set of measurements. The apparatus was routinely cleaned with water and ethanol following each session.

**Fear conditioning test.**

The auditory fear conditioning test was performed to assess the cognitive function and flexibility as described previously. [4](#_ENREF_4)^,^ [5](#_ENREF_5) On day 1 (conditioning phase), the conditioned stimulus was a 30 s tone (85 dB), and the unconditioned stimulus was a 5 s foot-shock (0.8 mA) that terminated simultaneously with the tone. This procedure was repeated four times at 1 min intervals. On day 2 (extinction phase), mice were placed in neutral cages, and after a lapse of 3 min, a 1 min tone (85 dB) was played six times at 1 min intervals without foot-shock. On day 3 (recall phase), the tone was played three times to test for recall of extinction learning (Milad and Quirk, 2002). Some of the mice underwent the recall phase (day 3) without undergoing the extinction phase (day 2) (No-Ext groups; No-Ext control and No-Ext PNE). Time spent freezing was automatically assessed by fear-conditioning video software (MED-VFC2-SCT-M; Med Associates Inc, USA) during the conditioning phase.

**Statistical analysis**

All data were shown as means ± standard error of the mean and analysed by Prism 8 (GraphPad software, USA). Data from the behavioural tests including three-chambered social interaction, PPI, locomotor, rotarod, EPM, novel object recognition and fear conditioning tests were analysed by two-way analysis of variance with or without repeated measures followed by *post hoc* Bonferroni test. Data from the PPI (acoustic startle response), locomotor (locomotor activity), rotarod (test phase), open field, marble burying, Y-maze and fear conditioning (contextual) tests were assessed using the Mann–Whitney *U* test.

**Cortical neuron cultures and time-lapse analysis**

Cortical neuron cultures were prepared as defined in previous studies [5](#_ENREF_5). Dissociated neurons were transferred to plates coated with poly-L-ornithine (Sigma-Aldrich). Time-lapse movies were captured using the IncuCyte ZOOM live-imaging system (Essen Bioscience, USA).[6](#_ENREF_6)

**II. Supplementary Figures**

**Figure S1** **Brain weight to body weight ratio of 3q29-del mice**

Five 8- to 9-week-old WT and 3q29-del male mice each measured and dissected. (a) Photographs of whole brains after perfusion fixation. Brains of wild-type mice on the left and 3q29-del mice on the right. (b) Comparison of body weights of wild-type and 3q29-del mice. Measured under anesthesia before perfusion fixation; comparison by Mann-Whitney t-test shows that 3q29-del weigh significantly less than WT (N=5, p=0.0159). (c) Comparison of post-fixation brain weights of WT and 3q29-del mice; comparison by Mann-Whitney t-test shows that 3q29-del brain weights are significantly less than WT (N=5, p=0.0079). (d) Brain:body weight ratio of WT and 3q29-del mice. No significant difference.

**Figure S2 3q29-del immunostaining of adult mouse brain with glial cell marker antibodies**

Histological analysis by fluorescent immunostaining of coronal brain sections from 3q29-del and WT 8-9 week old mice. The antibodies used were stained for CNP1 (a), a marker for mature oligodendrocytes, GFAP (b), a marker for astrocytes, and Iba1 (c), a marker for microglia. The resulting images were read by Fiji (ImageJ2) and divided into four regions: cortex, hippocampus, thalamus, and hypothalamus, and the signal per 1mm^2^ was quantified.

**Figure S3 3q29-del immunostaining of adult mouse brain with neuron specific marker antibodies**

Histological analysis by fluorescent immunostaining of coronal brain sections from 3q29-del and WT 8-9 week old mice. The antibodies used were stained for NeuN (a), Arc (b), or TH (c). The resulting images were also read by Fiji (ImageJ2) and divided into four regions: cortex, hippocampus, thalamus, and hypothalamus, and the signals per 1mm^2^ were quantified.

**Figure S4 Neurite outgrowth analysis of E15.5 cortical neurons by time-lapse observation**

Cortical neurons were aseptically prepared from E15.5 embryo's brains obtained by mating mature 3q29-del males with C57BL/6J females, and were cultured on dish and just observed for two days by Incucyte ZOOM. (a) Cell bodies (green) and neurites (red) were automatically recognized and quantified using the Neurotrack module. (b) Mean ± standard error of neurite length per view. (c) Mean ± standard deviation of the number of branches per view.

**Figure S5 General behavioral analysis of 3q29 deletion model male mice**

All behavioral analysis results, except those shown in main Figure 4. Data were obtained for eleven wild-type male and eleven 3q29-del male mice (a) Weight at 8~9 weeks of age measured before the start of the open field test. (b) Open field test: inner and outer distance, inner and outer duration, total distance, transition, jumping defecation, urination and grooming. (c) Y-maze test: total arm entry and alternation. (d) Elevated plus maze test: duration and number of entry. (e) Rota-rod test: training and test. (f) Social interaction test: sociability, social novelty and habituation. (g) Fear conditioning test: training, contextual, cue and pain threshold. A summary of the results is shown in Table 1.

**Figure S6 General behavioral analysis of 3q29 deletion model female mice**

Data were obtained for twelve wild-type female and twelve 3q29-del female mice (a) Weight at 8~9 weeks of age measured before the start of the open field test. (b) Open field test: inner and outer distance, inner and outer duration, total distance, transition, jumping defecation, urination, rearing and grooming. (c) Y-maze test: total arm entry and alternation. (d) Elevated plus maze test: head dip, time spent and number of entry. (e) Rota-rod test: training and test. (f) Social interaction test: sociability and social novelty. (g) Novel object recognition test: exploratory preference and total exploration time. (h) Prepulse inhibition test: Startle response and PPI. (i) Fear conditioning test and Pain threshold: training, cue, contextual pain threshold. ear conditioning test. A summary of the results is shown in Table 1.

**Figure S7 Individual data of each mouse for 24-hrs locomotion activity test**

Data for the individual mice comprising Figure 5. Horizontal axis shows 24-hour time axis and vertical axis shows activity per time of day. The average for wild-type mice is shown in the blue line graph and the average for 3q29-del mice is shown in the magenta line graph.

**Figure S8 Successive data on activity and body temperature in mice implanted with Nanotag in the abdominal cavity**

Data for the individual mice comprising Figure 6. Six weeks under the L-D cycle and two weeks switching to the D-D cycle for a total of eight consecutive weeks. Graphed with Nanotag Viewer software. The horizontal axis shows the 24-hour time axis, and the vertical axis shows the activity (histogram) and body temperature (line graph) for each day. Magenta arrows indicate the point of switching to the D-D cycle.

**III. Supplementary References**

1. Aida T, Chiyo K, Usami T, Ishikubo H, Imahashi R, Wada Y *et al.* Cloning-free CRISPR/Cas system facilitates functional cassette knock-in in mice. *Genome Biol* 2015; **16:** 87.

2. Schindelin J, Arganda-Carreras I, Frise E, Kaynig V, Longair M, Pietzsch T *et al.* Fiji: an open-source platform for biological-image analysis. *Nat Methods* 2012; **9**(7)**:** 676-682.

3. Serradas ML, Stein V, Gellner AK. Long-term changes of parvalbumin- and somatostatin-positive interneurons of the primary motor cortex after chronic social defeat stress depend on individual stress-vulnerability. *Front Psychiatry* 2022; **13:** 946719.

4. Sawahata M, Mori D, Arioka Y, Kubo H, Kushima I, Kitagawa K *et al.* Generation and analysis of novel Reln-deleted mouse model corresponding to exonic Reln deletion in schizophrenia. *Psychiatry Clin Neurosci* 2020; **74**(5)**:** 318-327.

5. Sekiguchi M, Sobue A, Kushima I, Wang C, Arioka Y, Kato H *et al.* ARHGAP10, which encodes Rho GTPase-activating protein 10, is a novel gene for schizophrenia risk. *Transl Psychiatry* 2020; **10**(1)**:** 247.

6. Arioka Y, Shishido E, Kubo H, Kushima I, Yoshimi A, Kimura H *et al.* Single-cell trajectory analysis of human homogenous neurons carrying a rare RELN variant. *Transl Psychiatry* 2018; **8**(1)**:** 129.
